# Tuberculous Orchitis Following Intravesical Bacille Calmette-Guérin (BCG) Therapy

**DOI:** 10.7759/cureus.2703

**Published:** 2018-05-29

**Authors:** Nikolai Klebanov, Aravind Raghavan

**Affiliations:** 1 Dermatology, Massachusetts General Hospital, Boston, USA; 2 Internal Medicine, Lahey Hospital and Medical Center, Burlington, USA

**Keywords:** tuberculosis, bacillus calmette-guerin (bcg), bladder cancer, bcg orchitis, testicular ultrasound, levofloxacin, rifampin, isoniazid, ethambutol, tuberculous orchitis

## Abstract

Intravesical therapy with Bacillus Calmette-Guérin (BCG) is a common and effective therapy for bladder carcinoma in situ. The risks associated with intravesical BCG therapy are significant and rare. Accurate diagnosis and prompt initiation of management significantly reduce the morbidity associated with these risks. Here, we discuss a case of BCG orchitis, a rare but treatable complication of intravesical BCG therapy. We present the case of a 55-year-old Puerto Rican incarcerated male who was diagnosed with high-grade Stage T1 urothelial carcinoma after presenting with hematuria, treated with transurethral resection of bladder tumor (TURBT), mitomycin, and intravesical BCG. He presented with left testicular pain and swelling after a failed course of ciprofloxacin with ultrasound findings characteristic of BCG orchitis. The patient received a combination therapy of levofloxacin, rifampin, isoniazid, and ethambutol, which resulted in symptom resolution. Combination therapy was initiated in this patient based on a high index of clinical suspicion, and in the absence of positive cultures. Competing diagnoses were considered and excluded based on the history, imaging findings, and observed response to therapy. As this is an uncommon diagnosis, and as routine infectious workup is often inconclusive, we emphasize that early anti-tuberculous treatment should be considered given a high degree of clinical suspicion based on history and patient presentation.

## Introduction

Treatment with intravesical Bacillus Calmette-Guérin (BCG) is an accepted and efficacious treatment modality for bladder cancer. BCG is an attenuated live vaccine derived from *Mycobacterium bovis* [1]. In preventing recurrence of the bladder cancer, intravesicular BCG has been reported to yield improved performance compared to epirubicin or doxorubicin, and a similar performance compared to mitomycin [2]. As an adjuvant therapy to transurethral resection of bladder tumor (TURBT), therapy with BCG both reduces recurrence rates and delays time to first recurrence when compared to TURBT alone [3].

However, intravesicular BCG treatment carries a risk of a variety of complications. In a study of 2,602 patients with superficial bladder cancer treated with several substrains of BCG, common complications included fever (2.9% of patients), hematuria (1.0%), granulomatous prostatitis (0.9%), lung or liver inflammation (0.7%), arthralgias (0.5%), epididymitis (0.4%), sepsis (0.4%), and multiple other symptoms and conditions [4]. A majority of the aforementioned complications are secondary to the immune upregulation due to the introduction of the BCG antigen. Among these complications, the most concerning is systemic sepsis or hypersensitivity, which is observed in one out of 15,000 patients treated with intravesical BCG.

With regard to local genitourinary complications, a retrospective cohort study of 256 patients who had undergone BCG therapy within a six-year period showed a 23.4% rate of local genitourinary complications, such as bladder involvement (5.9% of patients), penile lesions (5.9%), prostatitis (3.5%), and epididymo-orchitis (3.5%) [5]. The authors recommend a diagnostic and therapeutic algorithm for patients suspected to have a BCG infection as follows: If there is evidence of local manifestations, with or without high-grade fever and no evidence suggesting other diagnoses upon workup, it is recommended to start antituberculous therapy for six months and no further BCG instillations as part of therapy. Surgery and steroids may be considered as well if more prominent systemic symptoms are present [5].

Here, we describe a case of suspected BCG orchitis occurring in a patient five months after the last BCG treatment.

## Case presentation

A 55-year-old incarcerated male presented to the emergency room with a two-week history of left-sided scrotal pain and swelling.

This patient had a history of prostate cancer and high-grade urothelial bladder cancer. His prostate cancer was diagnosed 15 years prior to the current presentation, managed with radiation, and had since remained stable. The patient's high-grade, high-risk bladder cancer was diagnosed two years prior to current presentation. At the time, the patient initially presented with hematuria. A CT urogram performed at the time revealed a nonspecific bladder mass. Biopsy of the mass confirmed urothelial carcinoma. The patient's therapy course included a transurethral resection of the bladder tumor (TURBT), intravesical mitomycin, interferon alfa-2b, and intravesical BCG therapy. He received a total of seven intravesical injections of 50 mg live BCG per injection (350 mg cumulative dose) over the course of 18 months.

The patient's other significant medical history included rheumatoid arthritis, for which the patient was on weekly methotrexate and daily tofacitinib treatment. Both medications were discontinued on admission.

The patient developed scrotal pain two weeks prior to admission. He initially presented to the emergency department with this pain. At that time, he was diagnosed with acute bacterial epididymitis and was prescribed a course of ciprofloxacin. He had no improvement in his symptoms despite this treatment. Over the subsequent days, he reported experiencing intermittent chills and night sweats. He denied any penile discharge or any history of a sexually transmitted disease.

**Figure 1:**
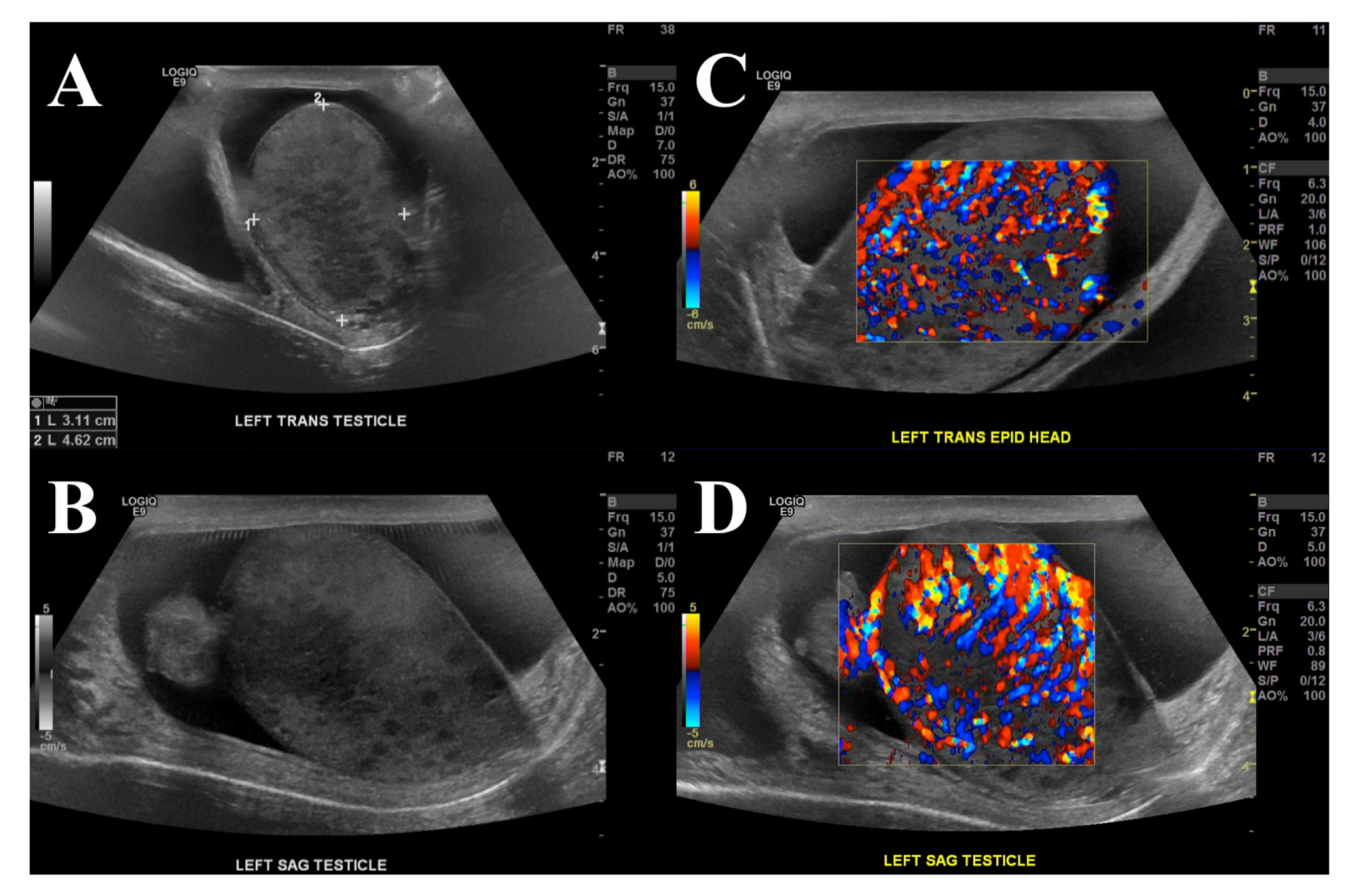
Diffuse hypoechogenicity and hypervascularity on scrotal ultrasound A-B) Ultrasonographic images of the left testis showing heterogeneous echogenicity with multiple patches of hypoechoic spots distributed throughout the testicle. C-D) Ultrasound with Doppler demonstrating marked hypervascularity of the left-sided structures. Patient also had a significant hydrocele surrounding the testicle.

Evaluation of the testicle with ultrasound revealed multiple diffuse nodular areas of hypoechogenicity (Figure 1A, 1B), as well as marked hypervascularity involving all of the left-sided structures (Figure 1C, 1D). Routine blood and urine cultures were negative, and mycobacterial blood and urine cultures, as well as bladder biopsy, were pending as of this writing.

Antituberculous therapy was started based on the following considerations regarding the patient's presentation. The lack of observed response to ciprofloxacin therapy decreased the likelihood of uncomplicated bacterial orchitis. We considered this patient's history of bladder cancer involving multiple BCG treatments and the increased likelihood of BCG orchitis. Possibly, his immunocompromised state secondary to the malignancy, in combination with mitomycin chemotherapy and immunomodulatory rheumatoid arthritis treatment, could have increased his susceptibility to a mycobacterial infection. Finally, as ultrasound findings were characteristic of BCG orchitis, the patient was promptly initiated on a combination therapy with levofloxacin, rifampin, isoniazid, and ethambutol. The patient showed marked improvement in pain control and testicular swelling over the following weeks.

## Discussion

In this particular case, the primary team employed a multidisciplinary approach with infectious disease and radiology consultations. Antituberculous treatment was initiated immediately upon admission primarily due to the high clinical suspicion for BCG orchitis. The patient’s local urological symptoms and characteristic ultrasound findings supported the diagnosis. Although laboratory studies were obtained, the treatment was initiated without awaiting results.

Microbiological workup included routine urine and blood cultures, which had remained negative throughout the admission. Mycobacterial blood and urine cultures, as well as bladder tissue biopsy, were pending at the time of this writing. A literature review revealed culture positivity of 41% in cases of tuberculous orchitis [5]. However, while 42% of tissue biopsy cultures were positive, only 24% and 5% of urine and blood cultures, respectively, had positive results. Often, negative culture results are obtained regardless of active *M. bovis* infection. Cultures are more likely to be negative in the early presentation of disease, and culture positivity also depends on a variety factors, including the count of organisms present as well as on specimen handling and culture technique [6]. Thus, the diagnosis of BCG orchitis infection relies primarily on observing a positive response to antituberculous treatment, as well as exclusion of other possible diagnoses.

In this case, we considered additional possible diagnoses. Bacterial orchitis secondary to ascending infection from gram-negative organisms was considered to be less likely, given the lack of response to a sufficient course of oral ciprofloxacin. Malignancy, such as a new primary tumor or a metastatic tumor from bladder cancer, was also included in the differential diagnosis. For instance, in one study, scrotal pain has been reported as the first presenting symptom in approximately 25% of testicular cancers [7]. The tender testicular swelling and the rapid two-week symptom onset increased the likelihood of an infectious, rather than neoplastic, etiology. Furthermore, the patient showed marked symptom improvement upon initiation of combination therapy with levofloxacin, rifampin, isoniazid, and ethambutol.

## Conclusions

Here, we discuss a case of BCG orchitis in a 55-year-old male who presented five months after the last intravesical BCG treatment cycle. On literature review, we identified that treatment for BCG orchitis with a combination of antituberculous therapy should be initiated, given a high index of clinical suspicion. Although no positive cultures were available on initiation of treatment, no competing diagnosis was considered as more likely. The patient's initial local urological symptoms improved significantly after treatment was administered. This case of a rare complication of intravesical BCG therapy supports the importance of a rapid initiation of antituberculous treatment, even with negative cultures. Clinicians should be aware of tuberculous orchitis as a rare but treatable BCG-associated condition.
